# Role of Acid Structure in Structure–Property Relationships of Reprocessable Epoxidized Soybean Oil Thermosetting Networks

**DOI:** 10.3390/polym18172180

**Published:** 2026-09-07

**Authors:** Madina Mussalimova, Ainash Baidullayeva, Alexey Shakhvorostov, Zhanserik Shynykul, Gaukhar Toleutay

**Affiliations:** 1 Department of Chemical and Biochemical Engineering, Geology and Oil-Gas Business Institute Named After K. Turyssov, Satbayev University, Almaty 050043, Kazakhstan; mussalimova.m@stud.satbayev.university (M.M.); shynykul.zhanserik@med-kaznu.com (Z.S.); 2 Department of Engineering Disciplines and Good Practices, Kazakh National Medical University Named After S.D. Asfendiyarov, Almaty 050012, Kazakhstan; 3 Institute of Polymer Materials and Technologies, Almaty 050019, Kazakhstan; a.shakhvorostov@ipmt.kz; 4 Department of Pharmaceutical Sciences, College of Pharmacy, The University of Tennessee Health Science Center, Memphis, TN 38103, USA; 5 Department of Chemistry, University of Tennessee, Knoxville, TN 37996, USA

**Keywords:** epoxidized soybean oil, bio-based vitrimer, transesterification, reprocessable thermosets, natural carboxylic acids

## Abstract

Thermosetting polymers offer excellent thermal stability, chemical resistance, and mechanical integrity, but their permanent covalent crosslinks limit recyclability and reprocessability. In this work, ESO-based vitrimer-like polyester networks were synthesized from epoxidized soybean oil (ESO) using tartaric, maleic, succinic, and tannic acids as catalyst-free curing agents. The influence of curing-agent structure on epoxy conversion, network homogeneity, thermal behavior, mechanical properties, chemical resistance, and reprocessing performance was systematically investigated. FTIR analysis indicated extensive epoxide ring opening and polyester network formation in all formulations. Tartaric and maleic acid systems exhibited stronger ester absorptions, higher gel content, and improved film uniformity, indicating more efficient network formation. The resulting materials showed good thermal stability, with degradation onset temperatures of 265–280 °C and maximum decomposition temperatures up to 400 °C. Mechanical performance strongly depended on acid structure: tannic acid produced the stiffest and strongest films, tartaric acid provided the best balance between strength and ductility, and succinic acid yielded less-uniform networks with reduced structural integrity. Reprocessing experiments demonstrated thermo-mechanical reprocessability consistent with vitrimer-like behavior in the tartaric- and maleic-acid-cured systems. These findings highlight curing-agent architecture as a key parameter for designing catalyst-free, renewable, and reprocessable ESO-based thermosets with tunable structure–property relationships.

## 1. Introduction

Thermosetting polymers are widely used in structural composites, adhesives, protective coatings, electronic encapsulation materials, and printed circuit boards because their permanently crosslinked three-dimensional networks provide high thermal stability, chemical resistance, mechanical strength, and structural integrity [1,2,3]. However, the irreversible covalent crosslinks that provide these advantages also prevent remelting and reshaping after curing, making conventional mechanical recycling and remolding impossible [1,4]. This permanent network topology further complicates the recovery of reinforcing fibers and other embedded components from composite structures and electronic assemblies [1,3]. Consequently, increasing attention has been directed toward recyclable thermosetting systems based on dynamic covalent chemistry and covalent adaptable networks (CANs), which enable bond exchange and network rearrangement [5,6,7,8].

Among CANs, vitrimers have attracted considerable attention because they combine the dimensional stability of thermosets with the reprocessability of thermoplastics. Their associative bond-exchange reactions allow network topology to reorganize while maintaining an approximately constant crosslink density during processing [9,10,11]. Consequently, these materials can undergo reshaping, repair, and thermo-mechanical reprocessing under appropriate conditions while largely preserving network integrity [9,12]. Vitrimer design has employed several dynamic chemistries, including transesterification, imine exchange, disulfide exchange, boronic ester exchange, and vinylogous urethane reactions [10,11,12,13]. These dynamic networks generally retain thermoset-like mechanical properties and chemical resistance at service temperatures while enabling stress relaxation, self-healing, and reprocessing at elevated temperatures [9,10,12]. Accordingly, they have been investigated for recyclable adhesives, coatings, structural composites, and functional electronic materials [11,13]. Bio-based vitrimer-like systems have recently attracted increasing attention as a strategy to combine recyclability with renewable feedstocks and improved sustainability of polymer materials. Among various bio-derived precursors, epoxidized soybean oil (ESO) has emerged as one of the most promising building blocks for vitrimer-like network formation due to its commercial availability, low toxicity, and high epoxy functionality originating from the epoxidation of unsaturated fatty acid chains [14,15,16]. The multifunctional triglyceride structure of ESO provides multiple reactive epoxy groups that can participate in ring-opening reactions with acids, alcohols, or amines, enabling the formation of crosslinked polymer networks with dynamic covalent characteristics [14,15]. Because soybean oil is produced at large industrial scale and can be readily chemically modified, ESO represents an attractive renewable platform for designing sustainable epoxy thermosets and vitrimer-like systems. Recent studies have demonstrated that ESO-based dynamic networks can exhibit promising properties such as self-healing capability, reprocessability, and closed-loop recyclability while maintaining adequate thermal stability and chemical resistance [15,16,17]. Consequently, ESO-derived vitrimer-like materials are increasingly considered as sustainable alternatives for coatings, adhesives, composite matrices, and recyclable polymer systems [14,16].

The performance of ESO-based thermosets and vitrimer-like materials strongly depends on the curing chemistry used to construct the polymer network. In epoxy–acid systems, curing typically proceeds through nucleophilic ring-opening reactions of epoxy groups with carboxylic acids, generating β-hydroxy ester linkages that form crosslinked polyester networks. These β-hydroxy ester linkages not only define the permanent network structure but may also enable thermally activated transesterification reactions, which can contribute to vitrimer-like network rearrangement under appropriate conditions. However, the extent to which such exchange processes are activated depends strongly on network structure and has not been systematically clarified for simple bio-based epoxy–acid systems. The architecture and properties of ESO networks are therefore governed by parameters such as crosslink density, curing agent functionality, and the structure of the hardener molecules. Previous studies have shown that fatty acids, polyacids, and polyphenolic curing agents can differently affect the rigidity, thermal stability, and mechanical performance of ESO-based thermosets [18], whereas catalytic systems may improve curing efficiency and network formation [19]. ESO-derived thermosets have also been reported to exhibit glass-transition temperatures of approximately 20–60 °C, depending on their formulation and curing conditions [20,21,22,23]. Thus, selecting appropriate curing agents and processing conditions is essential for tailoring the structure and properties of vegetable-oil-derived epoxy networks [24].

Despite the rapid development of ESO-based vitrimer-like systems, several important limitations remain in the current literature. One major challenge is that networks derived from soybean oil often exhibit relatively low stiffness and mechanical strength due to the flexible aliphatic structure of the triglyceride backbone. As a result, ESO-rich materials frequently show low glass transition temperatures and limited structural rigidity. For example, unmodified ESO-based systems have been reported with tensile strength as low as 2.8 MPa and Tg values around 16.9 °C, while some high-ESO polyurethane networks exhibit even lower mechanical strength and Tg values approaching −30 °C [25,26]. Although rigid co-monomers, fillers, and lignin-derived components can enhance network rigidity, these reinforcement strategies may increase formulation complexity [25,26,27,28,29]. Another recurring limitation is that many transesterification-based dynamic networks rely on catalysts or require relatively high processing temperatures to achieve practical bond-exchange rates, with reported topology-freezing temperatures ranging from 66 to 136 °C and activation energies of 73–104 kJ mol^−1^ depending on the formulation [12,30]. Moreover, some ESO-derived dynamic networks require remolding above 160 °C, frequently under pressure, potentially limiting the scalability and energy efficiency of reprocessing [30,31]. Consequently, achieving ESO-based vitrimer-like systems that simultaneously combine efficient curing, improved mechanical performance, and practical reprocessability remains a significant challenge in the development of sustainable thermosetting materials [26,32,33,34,35].

In this context, the molecular structure of the curing agent can be considered as a key design parameter controlling network formation, crosslink density, and potential dynamic rearrangement behavior in ESO-based systems. In this study, a comparative investigation of four natural acids, tartaric (TA), succinic (SU), maleic (MA), and tannic (TAN), was conducted to evaluate their performance as hardeners for ESO-based vitrimer-like polyester networks. Rather than treating these acids as interchangeable crosslinkers, this work examines how differences in molecular size, functionality, hydroxyl content, and rigidity influence epoxy conversion, network homogeneity, thermal behavior, mechanical performance, chemical resistance, and reprocessability. By systematically comparing these curing systems, the present work aims to clarify the relationships between hardener structure, network architecture, and the resulting functional properties of ESO-derived polymer networks. These correlations enable the rational design of catalyst-free, bio-based reprocessable thermosets.

## 2. Materials and Methods

### 2.1. Materials

Epoxidized soybean oil (ESO, epoxy value 6.3 mol per 100 g, average epoxy functionality ≈ 6.0, viscosity 250–350 mPa·s at 25 °C, density 0.99 g cm^−3^, CAS 8013-07-8) was used as the bio-based epoxy monomer without further purification. Tartaric acid (TA, MW 150.09 g mol^−1^, purity ≥ 99.5%, CAS 87-69-4) was used as a multifunctional diacid curing agent containing both carboxyl and hydroxyl groups. Succinic acid (SU, MW 118.09 g mol^−1^, purity ≥ 99%, CAS 110-15-6) was used as a biogenic linear dicarboxylic acid. Maleic acid (MA, MW 116.07 g mol^−1^, purity ≥ 99%, CAS 110-16-7) was used as an unsaturated dicarboxylic acid capable of promoting efficient esterification and network formation. Tannic acid (TAN, average MW ≈ 1700 g mol^−1^, purity ≥ 95%, CAS 1401-55-4) was used as a multifunctional polyphenolic curing component contributing to increased crosslink density. Ethyl L-lactate (purity ≥ 98%, CAS 97-64-3) was used as a green solvent for preparing acid solutions when required. All reagents were purchased from Sigma-Aldrich (St. Louis, MO, USA) and used as received without further purification.

### 2.2. Preparation of Ethyl L-Lactate Curing-Agent Solutions

Stoichiometric amounts of tartaric (TA), succinic (SU), maleic (MA), or tannic acid (TAN) were dissolved in ethyl L-lactate (R = 1) to form homogeneous solutions. The mixtures (0.72 g mL^−1^) were heated at 60–90 °C under magnetic stirring until complete dissolution (15–20 min).

### 2.3. Preparation of Thermosetting Resin

Epoxy formulations containing tartaric (TA), succinic (SU), or maleic acid (MA) were prepared at a COOH/epoxy molar ratio of 0.7, calculated using two carboxyl groups per acid molecule. Because tannic acid does not contain carboxyl groups, the ESO–TAN formulation was calculated separately using an OH/epoxy equivalent ratio of 0.7. The tannic acid equivalent was estimated using 25 phenolic hydroxyl groups per molecule and an average molecular weight of approximately 1701.2 g mol^−1^. In a typical procedure, ESO (2 g) was heated to 60 °C under stirring, followed by the gradual addition of the corresponding acid solution in ethyl L-lactate. The mixture was stirred at 60 °C for 15–20 min until a homogeneous resin was formed prior to curing. Thus, the ESO–TAN formulation was compared with the dicarboxylic-acid systems on a reactive-group equivalent basis rather than a COOH-equivalent basis.

### 2.4. Curing Procedure

The prepared resin mixtures were poured into Teflon molds and subjected to a stepwise thermal curing process [36]. Initially, the samples were heated at 65 °C under vacuum for 15 min to remove trapped air and residual solvent. The temperature was then increased to 105 °C and maintained for 2 h at atmospheric pressure to promote the epoxy–acid reaction. Finally, the samples were post-cured at 120 °C for 10–12 h to ensure complete conversion of epoxy groups and formation of fully crosslinked thermosetting networks.

### 2.5. Characterization

The chemical structure, thermal properties, mechanical performance, and chemical resistance of the prepared materials were characterized using standard analytical techniques.

### 2.6. Fourier Transform Infrared (FTIR) Spectroscopy

FTIR spectra were recorded on a Cary 660 FTIR spectrometer (Agilent Technologies, Santa Clara, CA, USA) in the range of 4000–700 cm^−1^ with a spectral resolution of 4 cm^−1^ and 16 scans in absorbance mode. The spectra were processed using Origin 2024 software (OriginLab Corporation, Northampton, MA, USA).

### 2.7. Thermogravimetric Analysis (TGA)

Thermogravimetric analysis was performed using a LabSys Evo thermogravimetric analyzer (Setaram, Caluire, France). Samples (8–10 mg) were heated from 30 to 600 °C at a heating rate of 10 °C min^−1^ under a nitrogen atmosphere. The thermograms were processed using OriginLab software (OriginLab Corporation, Northampton, MA, USA).

### 2.8. Tensile Testing and Mechanical Characterization

Mechanical properties were measured using a texture analyzer (TA.XT Plus, Stable Micro Systems, Godalming, UK) equipped with a tensile fixture. Rectangular specimens (ca. 20 mm × 20 mm) were used. The specimen thickness was approximately 0.20–0.23 mm, the effective width was 20 mm, and the gauge length between the grips was 10 mm. Approximately 5 mm of each specimen end was secured between the upper and lower tensile grips. The specimens were tested at room temperature at a constant crosshead speed of 2 mm s^−1^. Three independent specimens (*n* = 3) were tested for each mechanically testable formulation (ESO–TA, ESO–MA, and ESO–TAN), and the results are reported as mean ± standard deviation. The ESO–SU formulation was excluded because the cured films lacked sufficient structural integrity for reliable tensile testing. Because the rectangular specimen geometry did not conform to a standardized dog-bone configuration, the measurements were performed using an in-house comparative testing protocol rather than ASTM D638 [37] or ISO 527 [38]. Stress–strain curves were recorded, and tensile strength, Young’s modulus, and elongation at break were determined.

Young’s modulus was calculated by linear regression of the initial linear region of each stress–strain curve over a strain interval of 0–5%. Before fitting, strain values were converted from percentage to dimensionless strain by dividing by 100. The modulus was calculated as the slope of stress versus dimensionless strain and reported in MPa.

### 2.9. Conversion of Epoxide Groups

The degree of epoxide conversion (*α*, %) during curing was determined by monitoring the decrease in the characteristic epoxy absorption band in the FTIR spectra. For the ESO–MA formulation, epoxide conversion was evaluated at different curing times, and the final conversion parameter $\alpha_{0\to 12}$ was calculated after 12 h of curing. Conversion was calculated using the Beer–Lambert expression [39]:

(1) $$\alpha_{0\to t}(\%)=(1-\frac{{\left(A_{E}\right)}^{t}}{{\left(A_{E}\;\right)}^{0}})\;\times 100\;$$

where (*A_E_*)*^t^* and (*A_E_*)^0^ are the absorbances of the epoxy band at approximately 821 cm^−1^ before curing (*t* = 0) and after curing for time t, respectively.

No invariant reference band was used for spectral normalization. Therefore, the calculated conversion value was treated as a semi-quantitative estimate rather than a definitive absolute conversion value. All spectra were collected using the same instrumental settings and sample-contact procedure to minimize measurement-related variation.

### 2.10. Determination of Gel Content

Gel content was determined using a solvent extraction method. Pre-weighed cured samples (*m*_i_ ≈ 0.20 g) were immersed in tetrahydrofuran (THF) and dimethylformamide (DMF) for 24 h at room temperature under gentle agitation. After extraction, the samples were rinsed with fresh solvent and dried at 60 °C until constant mass (*m_d_*). The gel fraction was calculated as:

(2) $$Gel\;fraction\left(\%\right)=\frac{m_{d}}{m_{i}}\times 100$$

A higher gel fraction indicates a higher degree of crosslinking and better solvent resistance of the vitrimer-like network.

### 2.11. Chemical-Resistance Screening

Qualitative chemical-resistance screening was performed by immersing rectangular ESO-based film specimens (10 mm × 10 mm) in 0.1 M NaOH and 0.1 M HCl solutions at room temperature for 24 h. After immersion, the specimens were removed and gently blotted with filter paper. Changes in appearance, visible swelling, fragmentation, and partial dissolution were assessed visually. Quantitative swelling ratios, mass loss, soluble fractions, and surface morphology were not determined.

### 2.12. Reprocessing Test

Reprocessing ability was evaluated by mechanically fragmenting the cured films and placing the fragments into a Teflon mold. The material was hot-pressed at 120 °C for 10 min to form reprocessed films. Tensile testing was performed to evaluate the retention of mechanical properties after reprocessing.

## 3. Results

**Figure 1:**
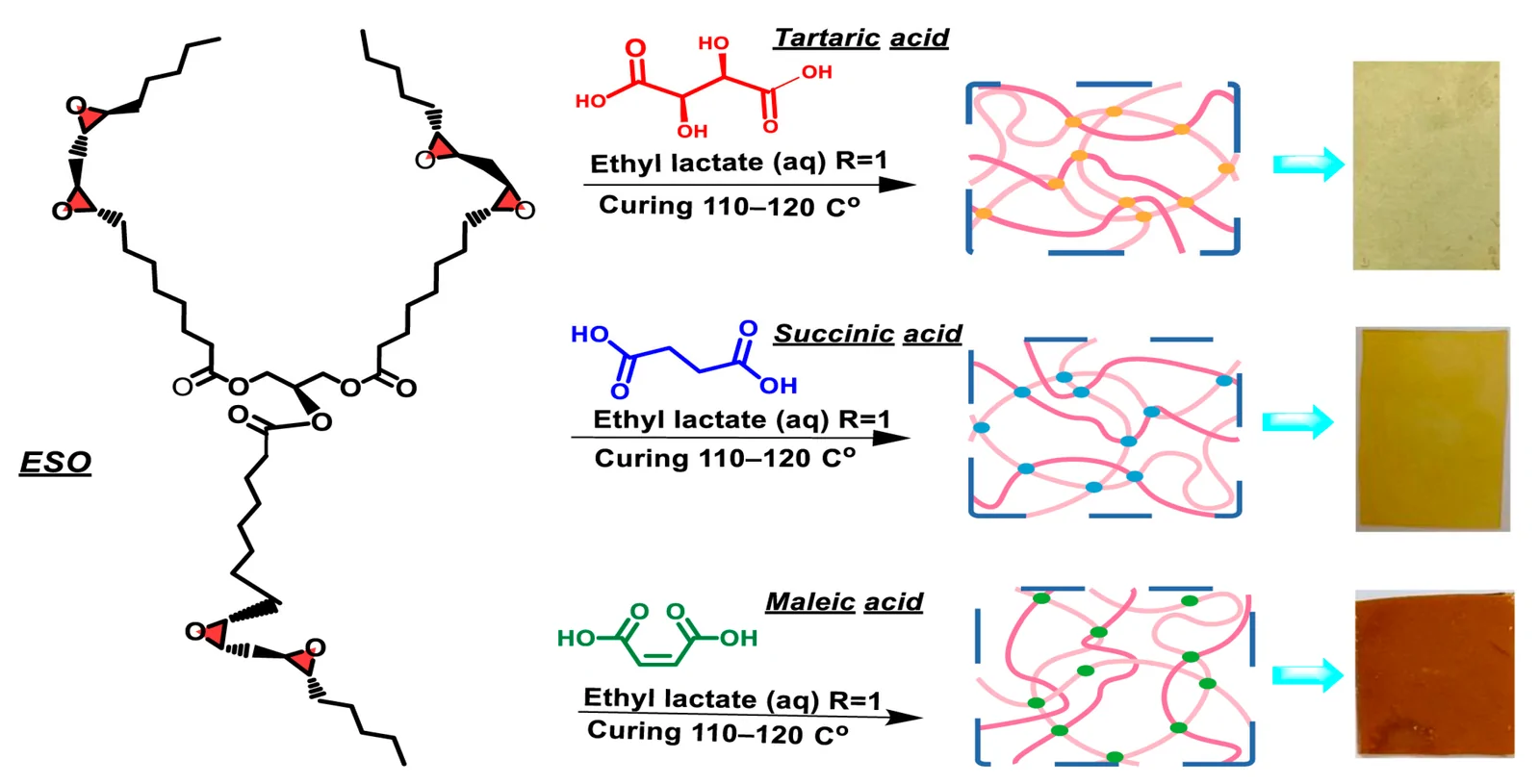
Formation of ESO-based vitrimer-like polyester networks using tartaric (TA), succinic (SU), and maleic (MA) acids. Pink curved lines represent ESO-derived polymer chains, while the colored dots indicate crosslinking sites formed by TA (orange), SU (blue), and MA (green). Cyan arrows indicate the resulting cured films. Curing was performed at 110–120 °C.

The formation of crosslinked networks in ESO-based systems was strongly governed by the molecular structure and functionality of the curing acids. Clear differences in mixing behavior, phase compatibility, and resulting film morphology were observed, indicating that the curing agent plays a critical role in determining network homogeneity. During the pre-curing stage, the ESO–MA formulation rapidly formed a homogeneous liquid phase without visible crystallization, suggesting efficient dissolution and good compatibility between maleic acid and the epoxy matrix. After curing at 110–120 °C, the resulting films were smooth, transparent, and optically uniform (Figure 1), indicating the formation of a relatively homogeneous polyester network. A similar behavior was observed for the ESO–TA system, although a more rapid increase in viscosity during heating was noted. This behavior can be attributed to the presence of both carboxyl and hydroxyl groups in tartaric acid, which promote epoxy ring opening and accelerate network formation. The cured ESO–TA films remained semi-transparent and flexible, consistent with a well-developed but more dynamic network structure. In contrast, the ESO–SU formulation exhibited reduced homogenization during mixing, particularly at temperatures below 80–90 °C. This resulted in cured films with lower transparency and visible heterogeneities across the surface, indicating incomplete dispersion of the curing agent and less uniform network formation.

**Figure 2:**
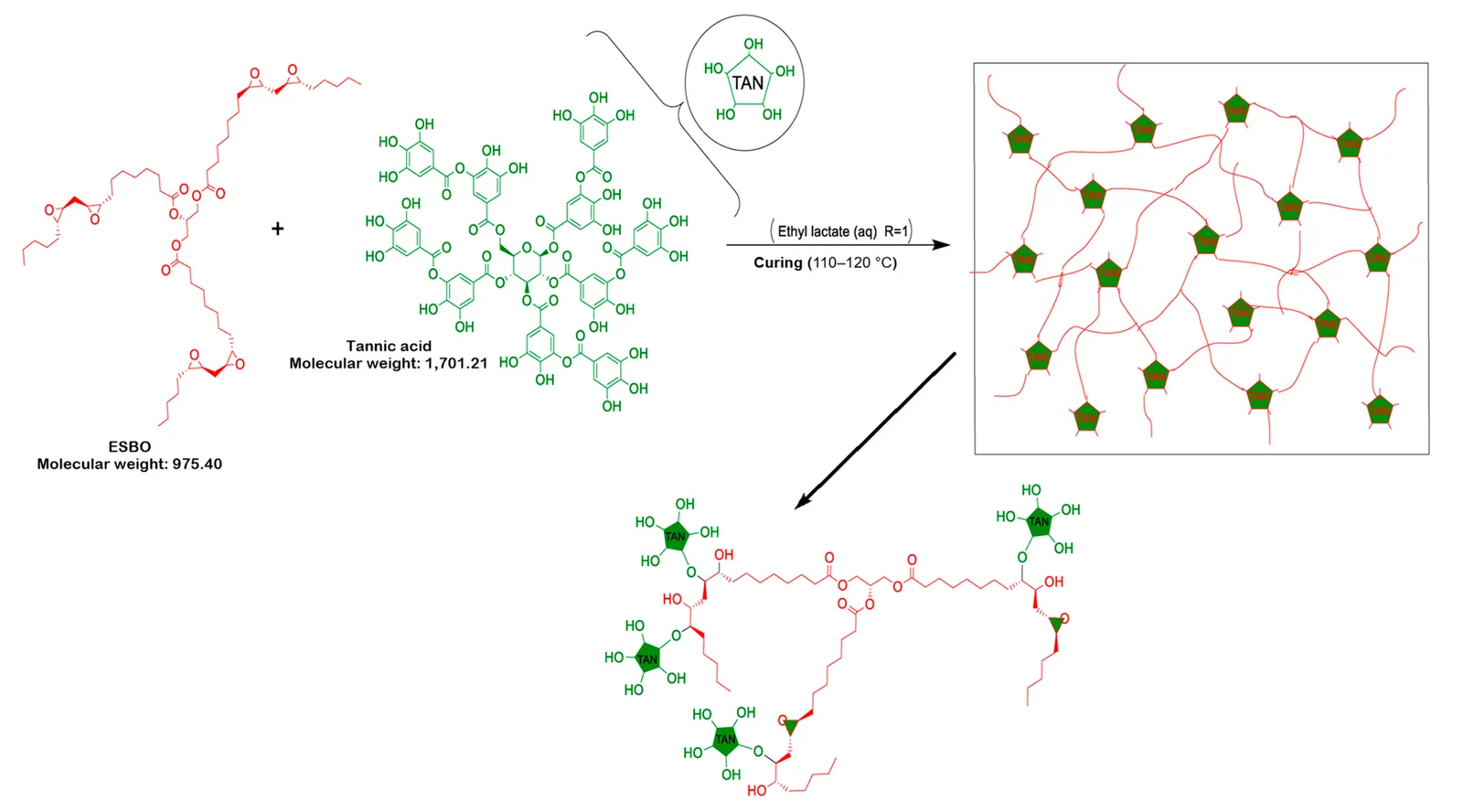
Chemical structures of ESO and tannic acid (TAN), proposed crosslinking mechanism in ethyl lactate (R = 1), schematic representation of the resulting polyester network, and optical appearance of the cured ESO-TAN film. The arrows indicate the curing process and the proposed formation of the crosslinked network. Red represents ESO-derived chains, while green represents TAN-derived crosslinking units.

A markedly different behavior was observed for the ESO–TAN system. During mixing, the incorporation of tannic acid resulted in early microphase separation, accompanied by localized viscosity increases and partial aggregation. After curing, the films appeared rigid and visually heterogeneous, with darker regions indicating a non-uniform distribution of tannic acid-rich domains within the polymer matrix (Figure 2). In contrast to the other curing systems, the ESO–TAN formulation exhibited the highest degree of morphological heterogeneity, suggesting that tannic acid strongly influences the curing behavior and final network organization.

**Figure 3:**
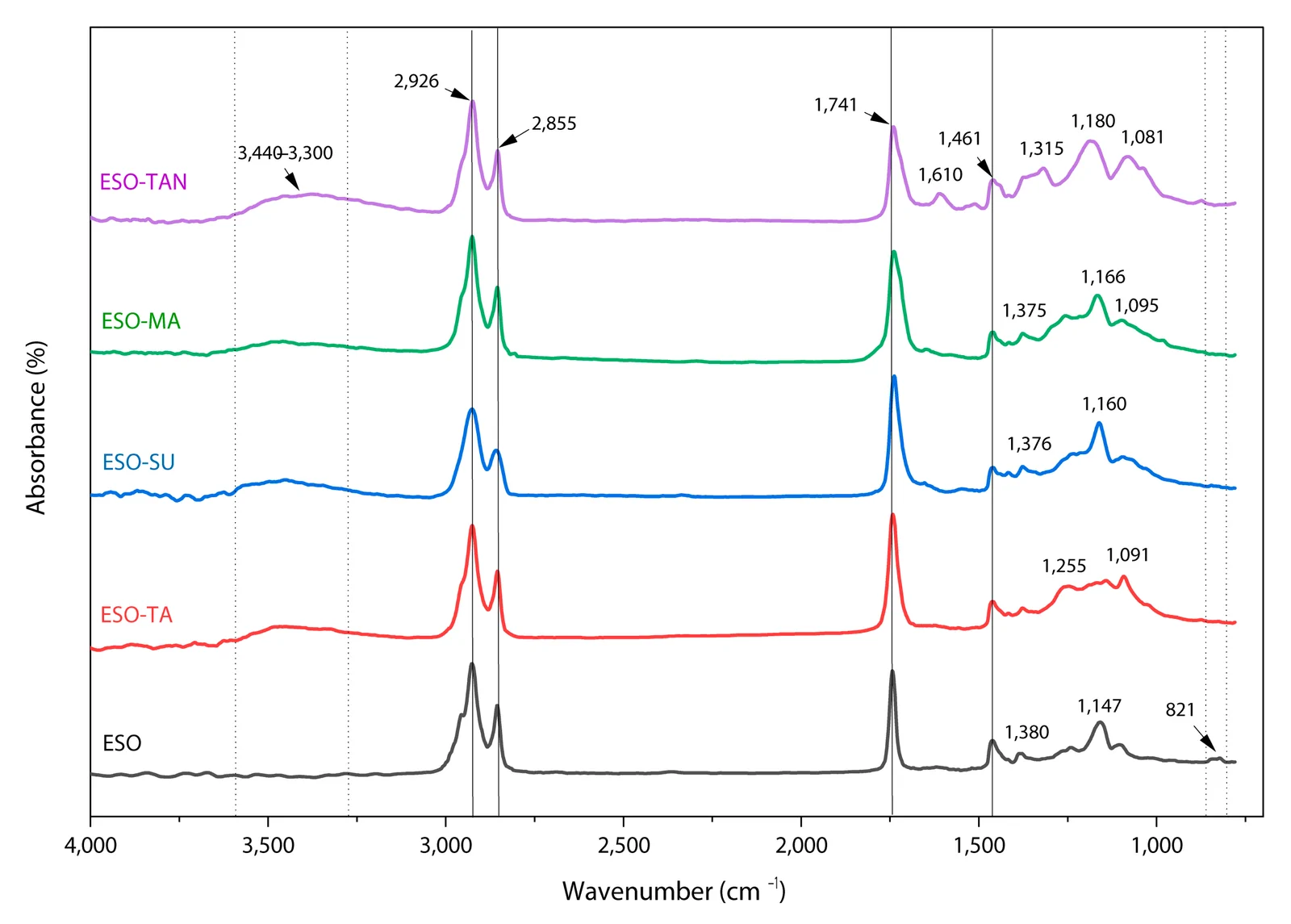
FTIR spectra of pristine epoxidized soybean oil (ESO) and ESO samples cured with tartaric (TA), succinic (SU), maleic (MA), and tannic (TAN) acids, showing the disappearance of the epoxy band (~821 cm^−1^) and the appearance of characteristic ester (1740–1730 cm^−1^), C–O–C (1260–1180 cm^−1^), and O–H (3440–3300 cm^−1^) absorption bands associated with polyester network formation.

To evaluate epoxide conversion and polyester network formation, FTIR spectra were recorded for pristine ESO and the corresponding acid-cured systems (Figure 3). The characteristic epoxy absorption band at ~821 cm^−1^, clearly observed in pristine ESO, disappeared after curing in all formulations, indicating extensive consumption of epoxy groups during curing. Concurrently, a strong carbonyl absorption band appeared in the 1743–1728 cm^−1^ region, consistent with ester bond formation in the cured networks. The intensity of the carbonyl and C–O–C absorption bands was higher for the ESO–MA and ESO–TA systems than for ESO–SU and ESO–TAN, suggesting differences in the extent of network formation among the curing systems. A broad O–H stretching band in the 3440–3300 cm^−1^ region was observed in all cured samples. The O–H band was most pronounced for the ESO–TA formulation. In the ESO–TAN spectrum, additional absorption bands in the 1600–1500 cm^−1^ region corresponding to aromatic skeletal vibrations were detected, confirming the presence of tannic acid-derived structural units within the cured material (Figure 3).

**Figure 4:**
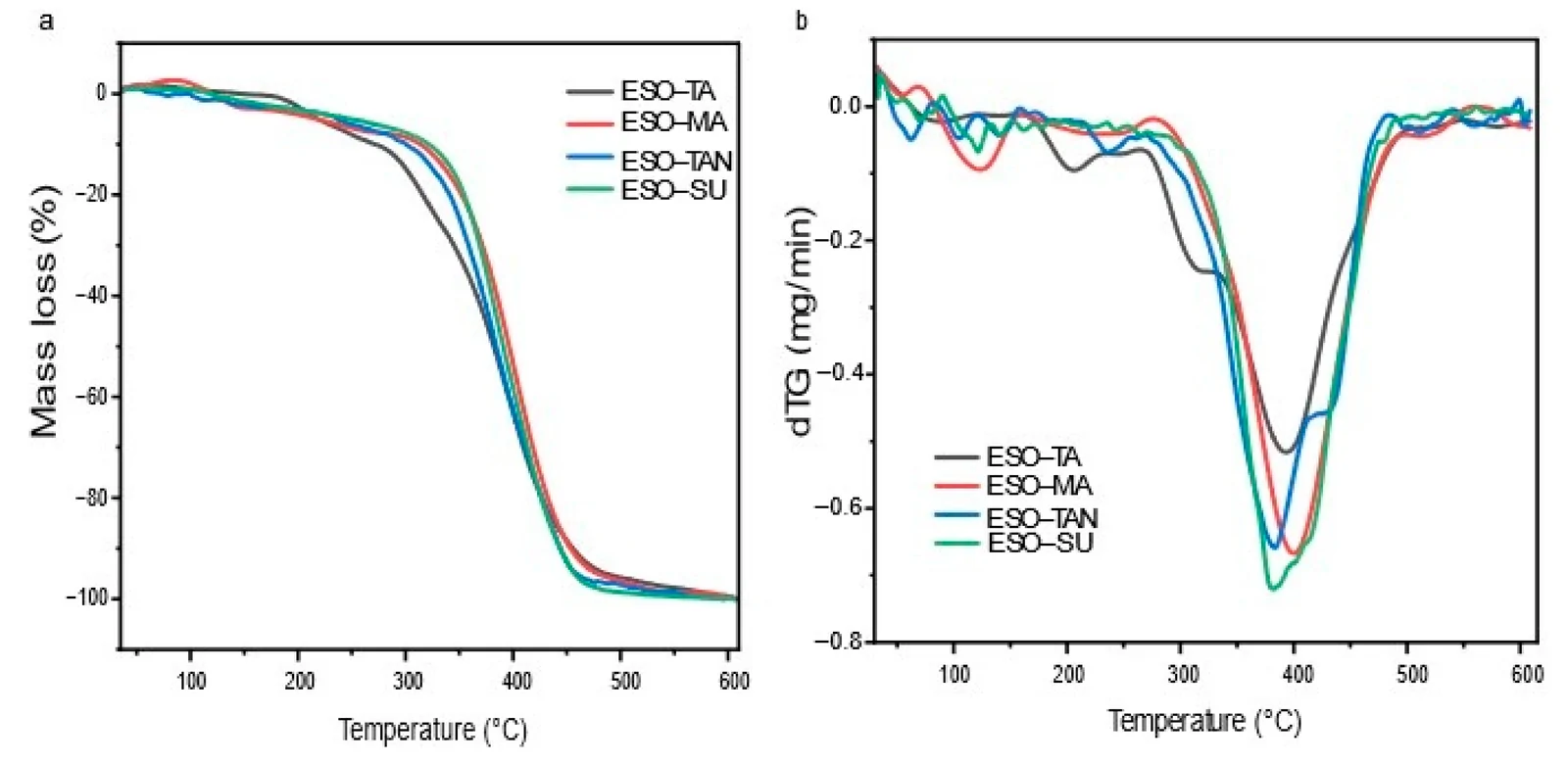
Thermogravimetric analysis (TGA) and derivative thermogravimetry (DTG) curves of ESO-based networks cured with tartaric (TA), succinic (SU), maleic (MA), and tannic (TAN) acids: (**a**) TGA curves showing mass loss as a function of temperature; (**b**) DTG curves showing the corresponding degradation rates.

To evaluate the thermal stability of ESO-based networks, thermogravimetric analysis (TGA) and derivative thermogravimetry (DTG) were performed for samples cured with different natural acids (Figure 4). All formulations exhibited a predominantly single-step degradation profile characteristic of the cured polymer networks. The onset of thermal degradation occurred within a relatively narrow temperature range (260–280 °C), while the main degradation event was observed between 360 and 400 °C, as indicated by the DTG curves. Differences among the curing systems were evident in both the degradation profiles and characteristic temperatures. ESO–MA and ESO–TA exhibited higher *T*_max_ values and narrower DTG peaks than ESO–SU and ESO–TAN. In contrast, ESO–SU showed a slightly lower degradation onset temperature and a broader DTG peak. The *T*_max_ values followed the order ESO–MA > ESO–TA > ESO–TAN > ESO–SU (Table 1).

**Figure 5:**
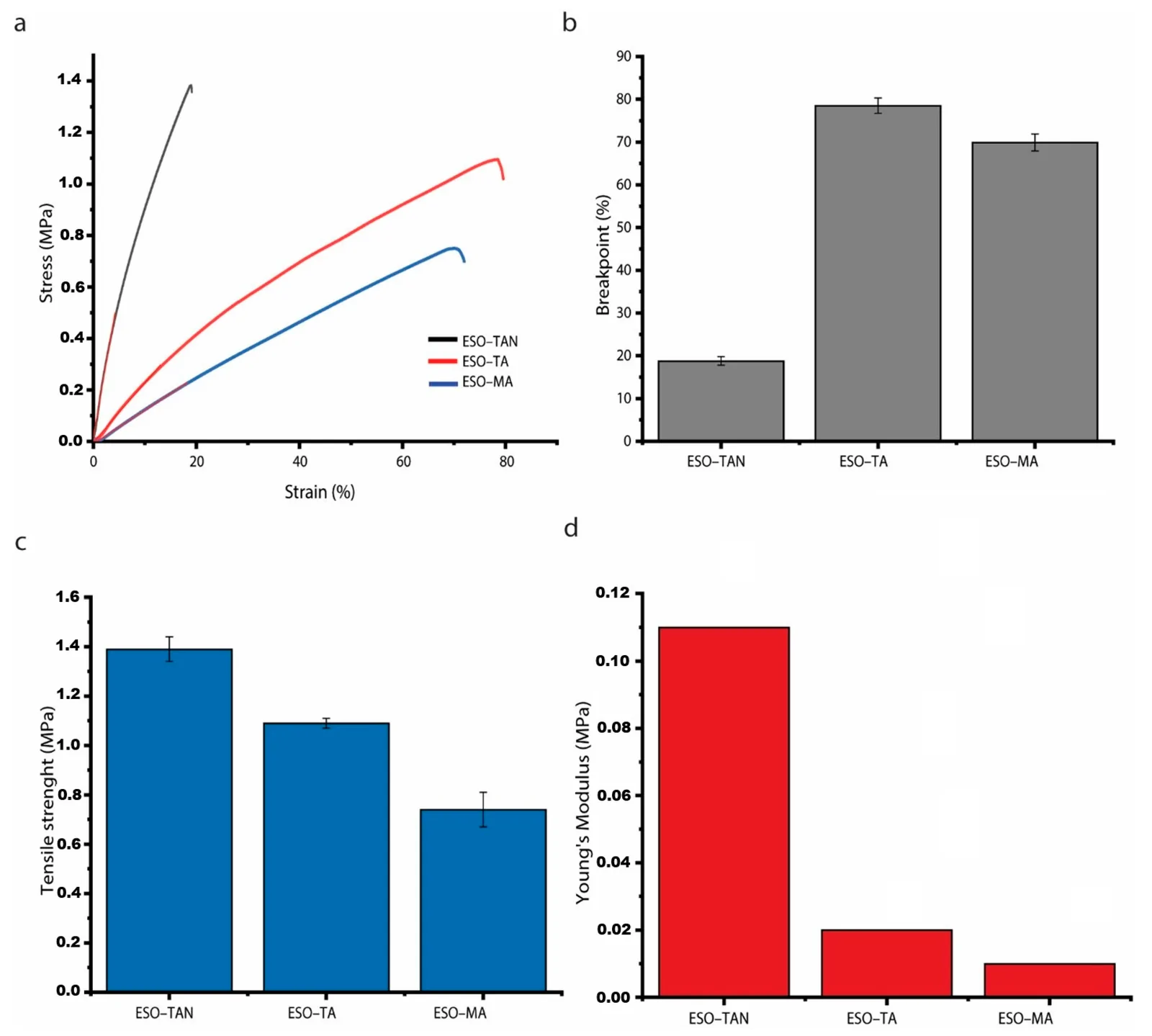
Mechanical performance of ESO-based vitrimer-like films cured with different acids (TA, MA, TAN): (**a**) stress–strain curves; (**b**) elongation at break; (**c**) tensile strength; (**d**) Young’s modulus.

The tensile properties of ESO-based films cured with different acids are shown in Figure 5 and summarized in Table 2. The ESO–SU system was excluded from mechanical testing because the cured films lacked sufficient structural integrity for reliable tensile measurements. The investigated formulations exhibited elastomer-like stress–strain behavior, although their mechanical responses differed substantially depending on the curing agent. ESO–TA showed the highest elongation at break (78.5%), followed by ESO–MA (69.9%), whereas ESO–TAN exhibited a markedly lower elongation (18.8%). In contrast, ESO–TAN displayed the highest tensile strength (1.39 MPa) and Young’s modulus (0.115 MPa), while ESO–MA exhibited the lowest tensile strength (0.74 MPa) and Young’s modulus (0.013 MPa). ESO–TA showed an intermediate tensile strength (1.09 MPa) together with the highest elongation at break.

**Figure 6:**
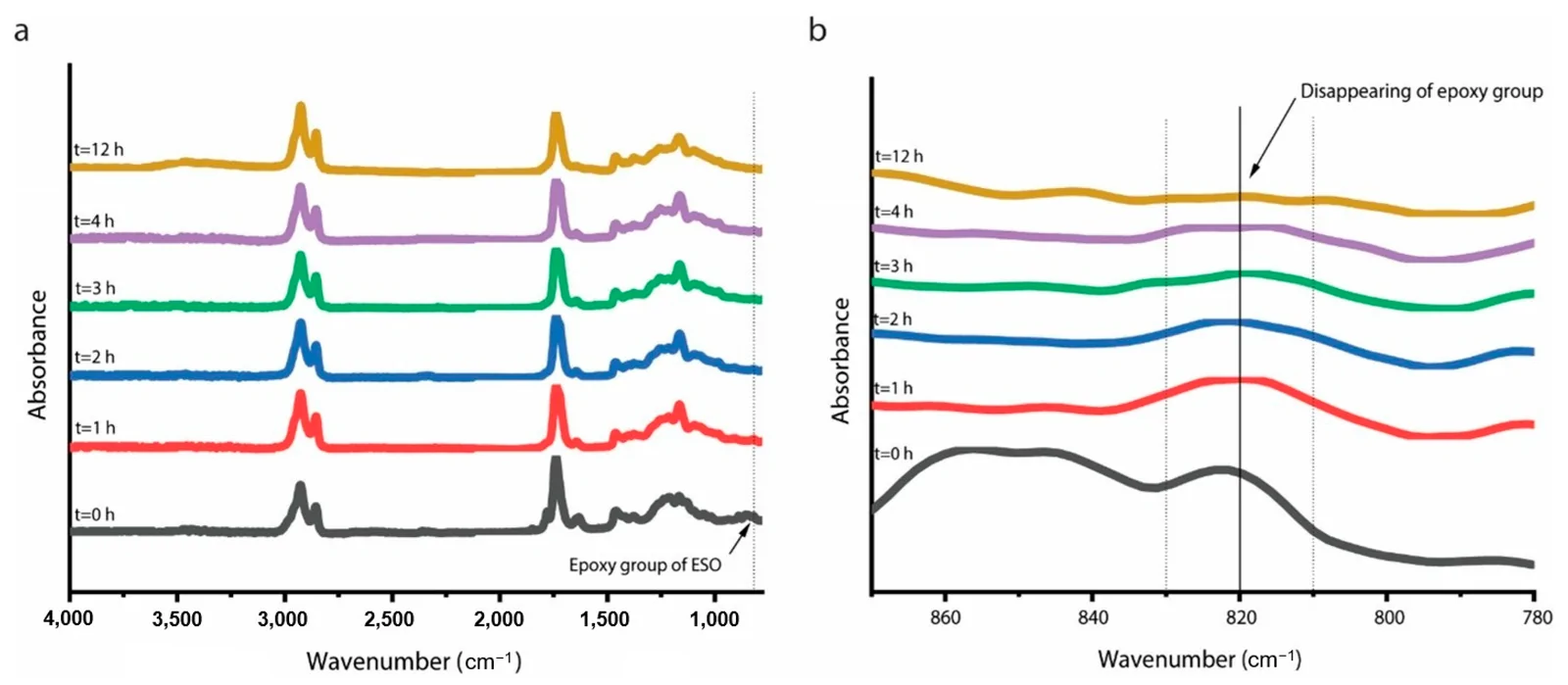
FTIR spectra of the ESO–MA system recorded at different curing times: (**a**) FTIR spectra in the 4000–1000 cm^−1^ region; (**b**) enlarged spectral region (870–780 cm^−1^) containing the oxirane absorption band at ~820–830 cm^−1^.

To monitor epoxy conversion during curing, FTIR spectra of the ESO–MA formulation were recorded at different reaction times (Figure 6a). The intensity of the characteristic oxirane band at ~820–830 cm^−1^ progressively decreased during curing, and the enlarged spectrum (Figure 6b) showed its near disappearance at the final reaction stage, indicating substantial consumption of epoxy groups. The calculated epoxide conversion parameter $\alpha_{0\to 12}$ for the ESO–MA formulation reached approximately 96% after 12 h of curing, as determined using Equation (1), demonstrating that most oxirane groups reacted during curing. Simultaneously, the intensity of the C–O and C–O–C absorption bands increased, consistent with the formation of β-hydroxy ester linkages during the curing process.

Gel content was determined after solvent extraction in THF and DMF to evaluate the insoluble fraction of the cured ESO-based networks (Figure S1, Table 3). All formulations exhibited substantial gel fractions after extraction, indicating the formation of insoluble polymer networks. The ESO–TA and ESO–MA systems showed the highest gel contents, reaching approximately 52.65% and 50.3% in DMF, respectively. The ESO–TAN formulation exhibited slightly lower gel contents (~47–48%), whereas the ESO–SU system showed the lowest values (~45–46%). DMF produced higher absolute gel-content values than THF for all formulations. However, the relative ranking differed between the solvents. In THF, the gel-content order was ESO–TA > ESO–SU > ESO–MA > ESO–TAN, whereas in DMF it was ESO–TA > ESO–MA > ESO–TAN > ESO–SU. This difference indicates that the measured gel content depended partly on the extraction solvent used.

**Figure 7:**
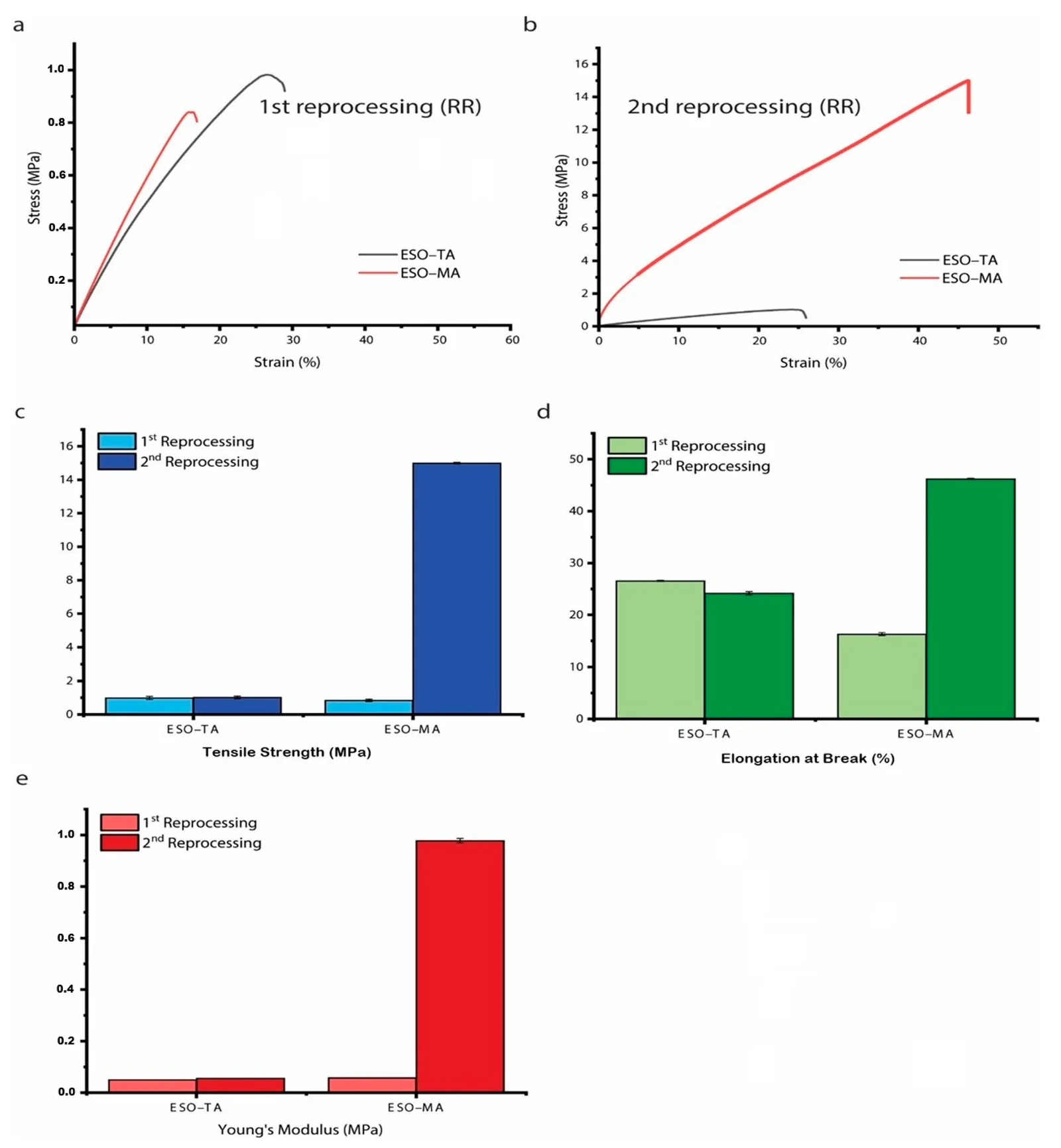
Mechanical performance of ESO–TA and ESO–MA vitrimer-like films after the first and second reprocessing cycles: (**a**) stress–strain curves after the 1st reprocessing; (**b**) stress–strain curves after the 2nd reprocessing; (**c**) tensile strength; (**d**) elongation at break; (**e**) Young’s modulus.

Qualitative chemical-resistance screening was performed by immersing the ESO-based films in 0.1 M NaOH and 0.1 M HCl solutions for 24 h (Figure S2). Exposure to NaOH caused visible swelling and partial dissolution, with the most pronounced changes observed for ESO–SU and ESO–TAN. In comparison, ESO–MA and ESO–TA retained their morphology to a greater extent (Figure S2a). All formulations remained visually intact after exposure to HCl under the investigated conditions (Figure S2b). Because quantitative swelling ratios and mass-loss measurements were not obtained, these results are interpreted only as qualitative observations. The reprocessing performance of the ESO-based vitrimer-like films was therefore evaluated over multiple hot-pressing cycles, and the results are summarized in Figure 7 and Table 4. The analysis focused primarily on the ESO–TA and ESO–MA formulations, since these systems previously exhibited the highest gel content and the most homogeneous network formation. After the first reprocessing cycle, both materials retained sufficient structural integrity to form continuous films under thermo-mechanical treatment at 120 °C for 10 min. As shown by the stress–strain curves in Figure 7, the ESO–TA sample preserved mechanical behavior close to that of the pristine material, with tensile strength of 0.983 ± 0.0946 MPa and Young’s modulus of 0.0502 ± 0.0001 MPa, while ESO–MA showed tensile strength of 0.842 ± 0.0689 MPa and Young’s modulus of 0.05806 ± 0.00009 MPa after the first reprocessing cycle (Table 4). These findings demonstrate that both networks remained amenable to thermo-mechanical reprocessing after curing. However, reprocessability alone does not confirm associative dynamic bond exchange.

A more distinct difference between the two formulations emerged after the second reprocessing cycle. The ESO–TA vitrimer-like network retained stable film morphology and showed only a slight increase in tensile strength from 0.98 to 1.02 MPa, accompanied by a small increase in Young’s modulus from 0.050 to 0.056 MPa and a moderate reduction in elongation at break from 26.6 to 24.2% (Table 4). This behavior indicates relatively stable mechanical performance during repeated thermo-mechanical reprocessing, with preservation of film integrity. In contrast, the ESO–MA vitrimer-like network exhibited increased tensile strength and stiffness after the second reprocessing cycle, reaching 15.0 ± 0.05 MPa and 0.98 ± 0.01 MPa, respectively, together with an elongation at break of 46.2%. The corresponding stress–strain curve also showed a marked change in mechanical response after the second cycle (Figure 7b). This change may reflect thermally induced network evolution; however, additional post-curing, densification, or further esterification during prolonged thermal treatment cannot be excluded.

**Figure 8:**
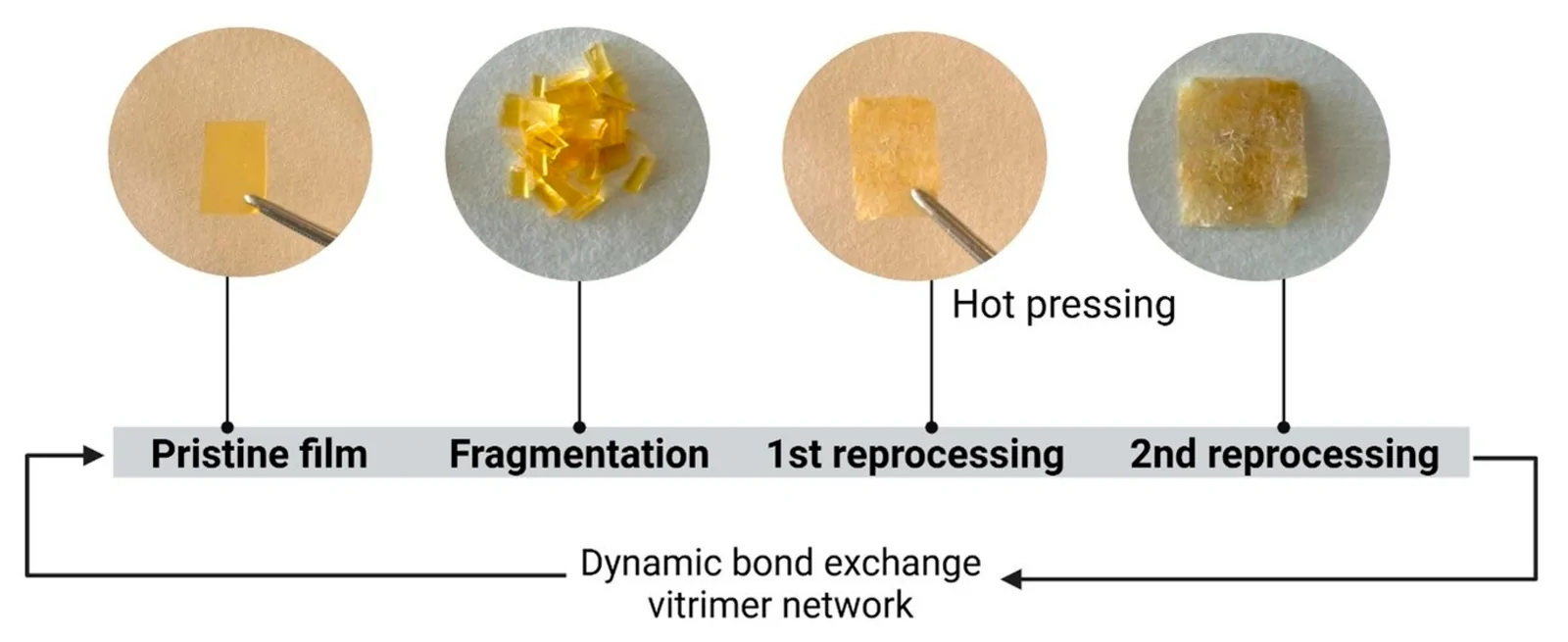
Reprocessing cycle of the ESO–MA vitrimer-like network: digital images of the pristine film, fragmented material, and films obtained after the first and second reprocessing cycles, together with a schematic illustration of the proposed thermally induced network rearrangement. The arrows indicate the successive fragmentation and hot-pressing cycles enabled by dynamic bond exchange within the network.

Overall, the reprocessing results show that the ESO-based vitrimer-like networks retain processability after repeated thermal treatment, although the long-term mechanical response depends strongly on curing-agent structure. ESO–TA shows more stable retention of film integrity, whereas ESO–MA undergoes more pronounced mechanical changes during reprocessing, likely accompanied by post-curing or densification effects. Visual observations (Figure 8) further confirm that the films maintain continuous morphology after reprocessing, despite slight color darkening and increased brittleness upon prolonged heating.

## 4. Overall Discussion and Conclusions

The distinct morphology and processing behavior of the different ESO systems can be correlated with the molecular characteristics of the curing agents. Maleic acid, owing to its relatively small molecular size and readily accessible carboxyl groups, is expected to promote efficient epoxy ring opening and more uniform network formation, consistent with previous reports [36,39]. Faster network formation also limits phase separation during curing, resulting in more homogeneous materials. Tartaric acid contains additional hydroxyl groups that may facilitate curing through hydrogen bonding and possible autocatalytic effects, contributing to more efficient network development [40]. In contrast, the lower compatibility of succinic acid with the hydrophobic ESO matrix likely slows its dissolution and may promote partial crystallization during processing, leading to a less homogeneous network structure [41,42]. The pronounced heterogeneity observed in the ESO–TAN system is consistent with the bulky polyphenolic structure of tannic acid, which can restrict molecular diffusion and reduce the accessibility of reactive groups during curing. These steric effects are expected to promote localized crosslinking and heterogeneous network formation rather than a uniformly distributed polymer network [43,44]. Overall, the results indicate that the molecular structure of the curing agent plays a key role in determining network topology, phase behavior, and morphological homogeneity in ESO-based vitrimer-like systems.

The FTIR results indicate that epoxy ring opening occurred efficiently in all formulations, consistent with the disappearance of the characteristic oxirane absorption band and the appearance of ester carbonyl signals, as widely reported for epoxide–acid curing systems [45,46]. Nevertheless, differences in the intensity of the carbonyl and C–O–C bands suggest that the efficiency of polyester network formation depends on the molecular structure of the curing acid [36,47]. The stronger ester-related absorptions observed for the ESO–MA and ESO–TA systems are consistent with more efficient esterification and a more homogeneous network structure. In contrast, the comparatively weaker ester absorptions in the ESO–SU and ESO–TAN systems suggest less efficient network formation and greater structural heterogeneity. The pronounced O–H absorption in the ESO–TA formulation is likely associated with both the hydroxyl groups inherently present in tartaric acid and those generated during epoxy ring opening. These hydroxyl groups may enhance intermolecular hydrogen bonding and facilitate transesterification reactions, contributing to improved network connectivity, in agreement with previous studies on dynamic polyester networks [40]. For the ESO–TAN system, the combination of aromatic absorption bands, broadened O–H stretching, and relatively weak ester signals is consistent with a more heterogeneous network structure. This behavior is likely associated with steric hindrance imposed by the bulky polyphenolic structure of tannic acid, which restricts molecular diffusion and reduces the accessibility of reactive groups during curing [43,44]. Overall, these findings demonstrate that, beyond complete epoxide conversion, the molecular architecture of the curing acid plays a critical role in determining the efficiency and uniformity of polyester network formation.

The similar degradation onset temperatures indicate that all curing systems produced thermally stable polyester networks with comparable resistance to the initial stages of thermal decomposition, consistent with previous studies on ESO-based thermosets [48,49]. However, the differences in *T*_max_, DTG peak shape, and residual char indicate that the molecular structure of the curing acid significantly influences the thermal degradation behavior of the resulting networks. The higher *T*_max_ values and narrower DTG peaks observed for the ESO–MA and ESO–TA systems are consistent with more efficient network formation and greater structural uniformity, as reported for maleic acid-based epoxy polyester networks [50]. In contrast, the broader DTG peak and lower degradation onset of ESO–SU suggest a less homogeneous network, which may be related to the greater flexibility of the aliphatic succinic acid structure and its influence on crosslinking efficiency [48]. Overall, these findings demonstrate that the chemical structure of the curing acid governs not only network formation but also the thermal degradation pathway and thermal stability of ESO-based vitrimer-like materials.

The mechanical behavior of the cured ESO networks is strongly influenced by the molecular structure of the curing agent. The high elongation observed for the ESO–TA and ESO–MA systems suggests that these networks retain greater chain mobility during deformation than the ESO–TAN network. In the case of tartaric acid, the presence of hydroxyl groups may contribute to intermolecular hydrogen bonding and influence network organization, resulting in greater deformability [36,40]. By contrast, the ESO–TAN formulation exhibited the highest tensile strength and stiffness but the lowest elongation, indicating the formation of a more rigid network. This behavior is consistent with the highly functional polyphenolic structure of tannic acid, which is expected to promote localized crosslinking and restrict polymer chain mobility [22]. Overall, these findings demonstrate that the balance between network rigidity and chain mobility is governed by the chemical structure and functionality of the curing agent, allowing the mechanical response of ESO-based vitrimer-like materials to be tuned through curing chemistry.

The present ESO-based networks exhibited tensile strengths of 0.74–1.39 MPa, elongations at break of 18.8–78.5%, and Young’s moduli of 1.30–11.5 MPa. These values partially overlap with those reported for ESO thermosets cured with hyperbranched Gly–SA crosslinkers, which exhibited tensile strengths of 0.183–2.323 MPa, elongations at break of 14.1–46.1%, and Young’s moduli of 1.5–7.2 MPa [51]. Histidine-accelerated ESO–tannic acid thermosets exhibited substantially higher tensile strengths and moduli of 14.7–23.0 MPa and 181–450 MPa, respectively, together with elongations at break of 6–35% [22]. Thus, the present catalyst-free networks showed mechanical properties within or near the range reported for flexible Gly–SA-cured ESO thermosets but were less stiff and strong than the histidine-accelerated ESO–tannic acid systems. Because the curing formulations, specimen geometries, and tensile-testing conditions differed among the studies, these comparisons should be considered qualitative.

The different behaviors of the four formulations can also be interpreted in terms of curing-agent functionality [26]. Tartaric acid contains two carboxyl groups and two hydroxyl groups; the latter may promote hydrogen bonding and facilitate epoxy-ring opening, contributing to efficient network formation and greater deformability of ESO–TA [40]. Maleic acid contains two carboxyl groups and an unsaturated, relatively rigid molecular structure, which may restrict rotational mobility and favor a more uniform network, although its carbon–carbon double bond was not demonstrated to participate directly in curing [36,39]. Succinic acid is a saturated, flexible aliphatic dicarboxylic acid without additional hydroxyl or unsaturated functionality. Its limited compatibility with the hydrophobic ESO matrix may contribute to less uniform curing and reduced structural integrity [24,41]. Tannic acid contains numerous phenolic hydroxyl groups and rigid aromatic units, which can increase stiffness and restrict chain mobility; however, its bulky structure and steric hindrance may limit reactive-group accessibility and promote heterogeneous network formation [21,22,43,44]. These structural differences are consistent with the observed variations in morphology, gel content, thermal stability, and mechanical behavior.

The time-dependent FTIR analysis demonstrates efficient curing of the ESO–MA system through the epoxy–acid esterification reaction. The progressive disappearance of the oxirane band together with the development of ester-related C–O and C–O–C absorptions is consistent with the formation of a crosslinked polyester network, as widely reported for epoxide–carboxylic acid systems [45]. The high epoxy conversion (~96%) indicates that the majority of reactive epoxy groups participated in network formation, suggesting that the selected curing conditions were sufficient to achieve an extensive degree of crosslinking. Such efficient network formation is consistent with the relatively simple molecular structure of maleic acid, whose readily accessible carboxyl groups facilitate epoxy ring opening and promote the formation of a homogeneous polymer network. This interpretation agrees with the FTIR, thermal, and mechanical results, which collectively indicate that the ESO–MA formulation undergoes efficient curing and develops a relatively uniform crosslinked structure.

Gel content provides an indirect measure of the extent of network formation and the fraction of polymer incorporated into the crosslinked structure. The higher gel contents observed for the ESO–TA and ESO–MA systems are consistent with more efficient curing and greater network connectivity, in agreement with the FTIR and thermal analyses. In contrast, the lower gel contents of the ESO–SU and ESO–TAN formulations suggest that a larger proportion of the material remained extractable, indicating less efficient network formation. For the ESO–SU system, this behavior may be associated with the lower reactivity and compatibility of succinic acid during curing, whereas the slightly lower gel content of ESO–TAN is consistent with the steric constraints imposed by the bulky polyphenolic structure of tannic acid, which can restrict the accessibility of reactive groups and promote localized rather than homogeneous crosslinking. The different gel-content rankings obtained in THF and DMF indicate that the measured extractable fraction depended partly on the extraction solvent. Because independent replicate measurements and variability estimates were not available, these data should not be interpreted as evidence of experimental reproducibility. These observations are consistent with previous reports showing that the molecular structure of the curing agent strongly influences crosslink density and network organization in ESO-based thermosetting systems [24,52].

The gel contents obtained in the present study (42.2–52.7%) were lower than those reported for highly crosslinked ESO thermosets. For example, histidine-accelerated ESO–tannic acid thermosets exhibited gel fractions of 83.1–88.2% [22]. The lower values obtained here indicate that a substantial fraction of the material remained extractable, potentially including unreacted components, soluble oligomers, and polymer chains that were not incorporated into the permanent network. This behavior may be associated with the catalyst-free curing conditions, the reactive-group/epoxy ratio of 0.7, and differences in curing-agent compatibility and accessibility. However, direct quantitative comparison should be made cautiously because the published study used Soxhlet extraction with acetone for 48 h, whereas the present study employed static extraction in THF and DMF for 24 h. Therefore, the measured gel contents confirm the presence of an insoluble network but do not indicate complete incorporation of all components or a highly crosslinked structure.

The qualitative responses of the ESO-based networks to alkaline and acidic media may reflect differences in their network structures. Polyester networks containing β-hydroxy ester linkages are susceptible to alkaline hydrolysis through ester-bond cleavage [24,25]. The greater visible swelling and partial dissolution of ESO–SU and ESO–TAN suggest lower morphological retention under the investigated alkaline conditions. The hydrolysable character of tannic acid has also been demonstrated previously, with thermal and hydrolytic treatments causing its partial or complete conversion into lower-molecular-weight phenolic compounds [53]. Although such conversion was not directly evaluated in the present study, this behavior may be relevant when interpreting the qualitative response of ESO–TAN to alkaline exposure. ESO–MA and ESO–TA retained their visible morphology to a greater extent, whereas all formulations remained visually intact following exposure to 0.1 M HCl. However, because quantitative swelling, mass-loss, and soluble-fraction data were not obtained, these observations do not permit quantitative comparison of chemical resistance among the formulations.

The reprocessing behavior of the present networks supports vitrimer-like rearrangement through exchangeable β-hydroxy ester bonds, although the relatively long treatment time required at 120 °C suggests slower bond-exchange kinetics than in many catalyst-assisted epoxy vitrimers [54,55,56,57]. Within this catalyst-free system, the difference between ESO–TA and ESO–MA highlights the importance of curing-agent structure in controlling thermo-mechanical reprocessing behavior. The TA-cured network retained film integrity and mechanical stability more consistently over repeated cycles, whereas the MA-cured network showed a stronger mechanical increase after the second cycle, suggesting that network rearrangement during reprocessing was accompanied by additional post-curing, densification, or further esterification. This contrast is consistent with the molecular features of the hardeners: tartaric acid contains both carboxyl and hydroxyl groups, which may promote more controlled associative exchange through internal activation and hydrogen-bond-assisted rearrangement, while MA may favor secondary structural changes during prolonged heating [12,58]. Such behavior agrees with previous reports showing that hydroxyl content, polyacid functionality, and network mobility strongly influence transesterification efficiency and long-term mechanical retention in catalyst-free polyester vitrimer systems [5,59].

Several limitations of the present study should be acknowledged. Thermal characterization was limited to TGA; therefore, glass-transition temperatures and temperature-dependent network mobility were not established by DSC or DMA. Thermo-mechanical reprocessing did not directly confirm associative dynamic bond exchange because temperature-dependent stress-relaxation measurements and activation-energy analysis were not performed. Furthermore, network homogeneity and crosslink connectivity were inferred from visual appearance, FTIR spectra, and gel-content measurements rather than directly established using microscopy, morphological imaging, DMA, or swelling-based crosslink-density analysis. Consequently, the proposed effects of phase separation, molecular diffusion, steric hindrance, and localized crosslinking should be regarded as plausible interpretations rather than directly demonstrated mechanisms. Future DSC, DMA, stress-relaxation, microscopy, and quantitative swelling studies are required to verify these interpretations.

In summary, fully ESO-based vitrimer-like polyester networks were successfully synthesized by curing ESO with natural organic acids (TA, MA, SU and TAN). The results demonstrate that the molecular structure and functionality of the curing agent strongly affect epoxy conversion, network homogeneity, gel content, thermal stability, mechanical response, chemical resistance, and reprocessing behavior of the resulting materials. Among the investigated systems, the TA- and MA-based formulations exhibited the most efficient network formation, as evidenced by their higher gel content, stronger ester-related FTIR absorptions, greater morphological retention during qualitative alkaline-resistance screening, and more homogeneous film morphology. The prepared networks showed good thermal stability, with degradation onset temperatures in the range of 265 to 280 °C and maximum decomposition temperatures up to 400 °C. Mechanical behavior could be tuned by the choice of curing acid, with TAN affording the stiffest and strongest material, TA providing the best balance between strength and ductility, and SU producing less-uniform networks with reduced structural stability. Repeated hot-pressing demonstrated thermo-mechanical reprocessability consistent with vitrimer-like behavior, particularly for the ESO–TA and ESO–MA systems; however, associative dynamic bond exchange was not directly confirmed. The ESO–TA network exhibited more stable mechanical retention during repeated reprocessing, whereas the ESO–MA system showed more pronounced network rearrangement, likely accompanied by post-curing or densification during thermal treatment. With the combination of renewable feedstock origin, catalyst-free curing, tunable thermo-mechanical behavior, chemical stability, and reprocessability, these ESO-based vitrimer-like materials represent promising candidates for the development of reprocessable ESO-based thermosetting materials.

## Figures and Tables

**Table 1:** Thermal degradation parameters of ESO-based networks cured with different natural acids.

Sample	*T*_onset_	*T*_max_ (°C)
ESO–MA	280	400
ESO–TA	275	388
ESO–TAN	268	382
ESO–SU	265	370

**Table 2:** Mechanical properties of ESO-based vitrimer-like films cured with TAN, TA, and MA.

Sample	Tensile Strength (MPa)	Elongation at Break (%)	Young’s Modulus (MPa)
ESO–TAN	1.39 ± 0.03	18.8 ± 0.5	0.115 ± 0.0004
ESO–TA	1.09 ± 0.02	78.5 ± 1.2	0.0231 ± 0.00003
ESO–MA	0.74 ± 0.01	69.9 ± 1.0	0.0130 ± 0.00001

**Table 3:** Gel content of ESO-based vitrimer-like networks after solvent extraction in THF and DMF.

Sample	Residual Mass (g, THF)	Residual Mass (g, DMF)	Gel Content (%, THF)	Gel Content (%, DMF)
ESO–TAN	0.084	0.096	42.2	47.9
ESO–MA	0.088	0.101	43.8	50.3
ESO–TA	0.098	0.105	49.0	52.7
ESO–SU	0.091	0.093	45.4	46.4

**Table 4:** Mechanical properties of ESO–TA and ESO–MA vitrimer-like films after the first and second reprocessing cycles.

Property	ESO–TA (1st)	ESO–MA (1st)	ESO–TA (2nd)	ESO–MA (2nd)
Tensile strength, MPa	0.98 ± 0.09	0.84 ± 0.07	1.02 ± 0.08	15.0 ± 0.05
Elongation at break, %	26.6 ± 0.1	16.3 ± 0.3	24.2 ± 0.3	46.2 ± 0.1
Young’s modulus, MPa	0.050 ± 0.0001	0.058 ± 0.0001	0.056 ± 0.0002	0.98 ± 0.01

## Data Availability

The original contributions presented in this study are included in the article/supplementary material. Further inquiries can be directed to the corresponding authors.

## Supplementary Materials

The following supporting information can be downloaded at: https://www.mdpi.com/article/10.3390/polym18172180/s1, Figure S1: Visual appearance of ESO-based vitrimer-like samples after solvent extraction: (a) THF; (b) DMF. The remaining insoluble fractions correspond to the gel content of networks cured with TAN, MA, TA, and SU; Figure S2: Chemical resistance of ESO-based vitrimer-like films after immersion in aqueous solutions: (a) NaOH (0.1 and 1 M); (b) HCl (0.1 and 1 M). Samples correspond to networks cured with MA, TA, SU, and TAN.

## Abbreviations

The following abbreviations are used in this manuscript:

ESO	Epoxidized soybean oil
TA	Tartaric acid
SU	Succinic acid
MA	Maleic acid
TAN	Tannic acid
FTIR	Fourier transform infrared
TGA	Thermogravimetric analysis
DTG	Derivative thermogravimetry
THF	Tetrahydrofuran
DMF	Dimethylformamide
HCl	Hydrochloric acid
NaOH	Sodium hydroxide
