# Supplementary material for: Role of Acid Structure in Structure–Property Relationships of Reprocessable Epoxidized Soybean Oil Thermosetting Networks
Source: Polymers (Basel). 2026 Sep 7;18(17):2180. doi: 10.3390/polym18172180 (PMC13568364; doi:10.3390/polym18172180)
Supplement: Supplementary file 1 [file polymers-18-02180-s001.zip › polymers-4507148-supplementary.pdf]

Role of Acid Architecture in Structure-Property Relationships of Dynamically Crosslinked Epoxidized Soybean Oil Vitrimers

Figure S1. Visual appearance of ESO-based vitrimer-like samples after solvent extraction: (a) THF; (b) DMF. The remaining insoluble fractions correspond to the gel content of networks cured with TAN, MA, TA, and SU.

Figure S2. Chemical resistance of ESO-based vitrimer-like films after immersion in aqueous solutions: (a) NaOH (0.1 and 1 M); (b) HCl (0.1 and 1 M). Samples correspond to networks cured with MA, TA, SU, and TAN.

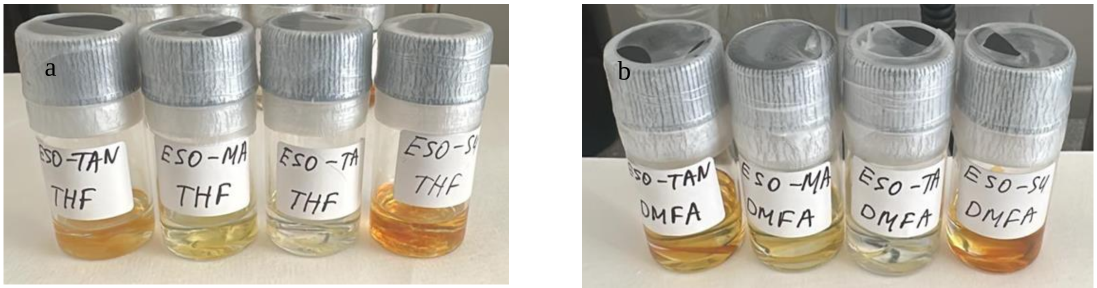

Figure S1. Visual appearance of ESO-based vitrimer-like samples after solvent extraction: (a) THF; (b) DMF. The remaining insoluble fractions correspond to the gel content of networks cured with TAN, MA, TA, and SU.

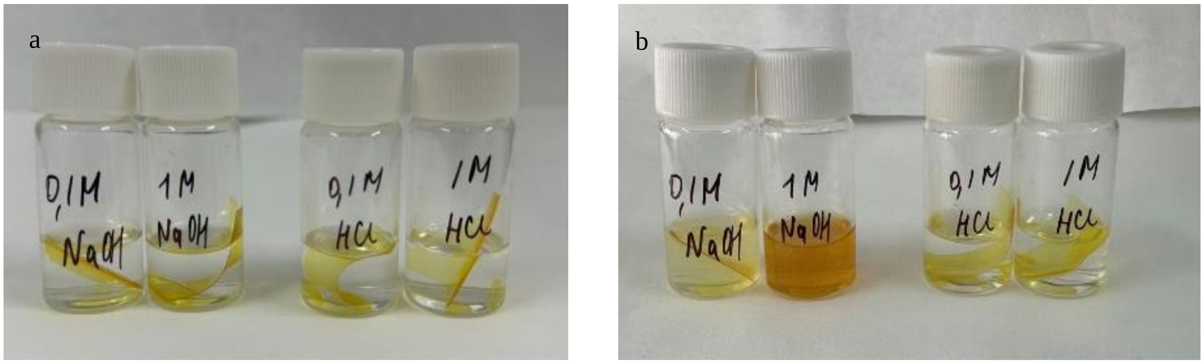

Figure S2. Chemical resistance of ESO-based vitrimer-like films after immersion in aqueous solutions: (a) NaOH (0.1 and 1 M); (b) HCl (0.1 and 1 M). Samples correspond to networks cured with MA, TA, SU, and TAN.
